# Supplementary figures and images for: Disrupting hierarchical control of nitrogen fixation enables carbon-dependent regulation of ammonia excretion in soil diazotrophs
Source: PLoS Genet. 2021 Jun 10;17(6):e1009617. doi: 10.1371/journal.pgen.1009617 (PMC8219145; doi:10.1371/journal.pgen.1009617)

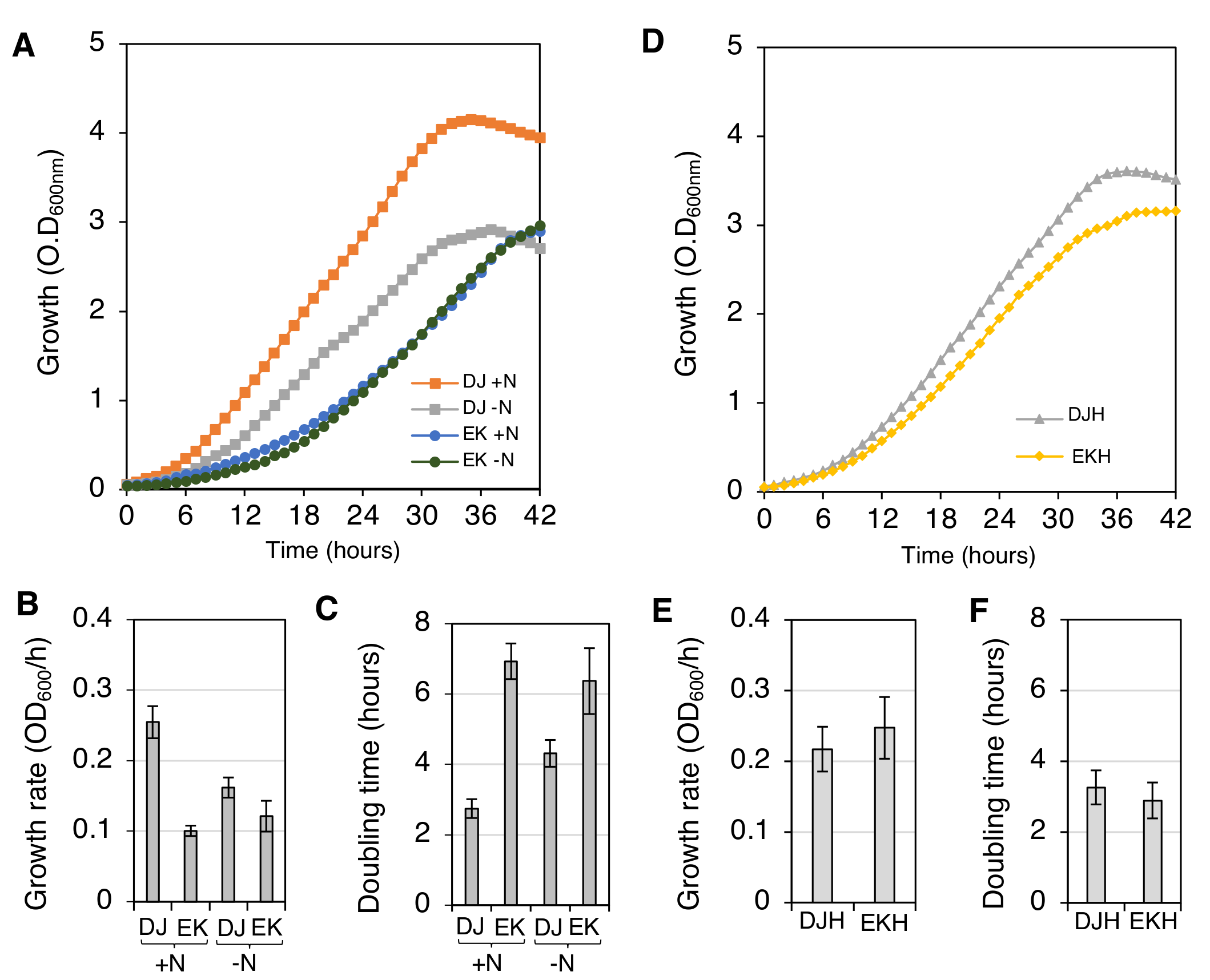

Supplement: S1 Fig — (A) Strains were grown in MBB media supplemented with 2% sucrose either in the presence (+N) or absence (-N) of 25 mM ammonium acetate. The growth rates (B) and doubling times (C) in the exponential phase of growth are also shown. (D) Strains carrying a nifH insertion were grown only in the presence of 25 mM ammonium acetate, given that they are unable to grow diazotrophically. The growth rates (E) and doubling times (F) in the exponential phase of growth were calculated from the data in (D). Cells were assayed for growth on a 24-well microplate (Greiner-Bio one #662160) using the Biotek EON plate reader as described in the Materials and Methods section. The absorbances recorded at 600 nm were corrected to a pathlength of 1 cm. (TIF) [file pgen.1009617.s001.tif]

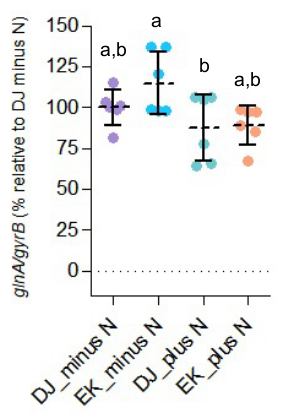

Supplement: S2 Fig — The strains were grown in minimal media supplemented with 2% sucrose under diazotrophic conditions (-N) or in the presence of excess ammonium chloride (+N). Data is relative to the maximum level of detected transcripts in the wild type under diazotrophic conditions (DJ -N) estimated from absolute quantification. The data is representative from 2 independent RNA purifications performed in technical triplicate. Plots followed by different letters are statistically different according to ANOVA with post-hoc Tukey’s HSD. (TIF) [file pgen.1009617.s002.tif]

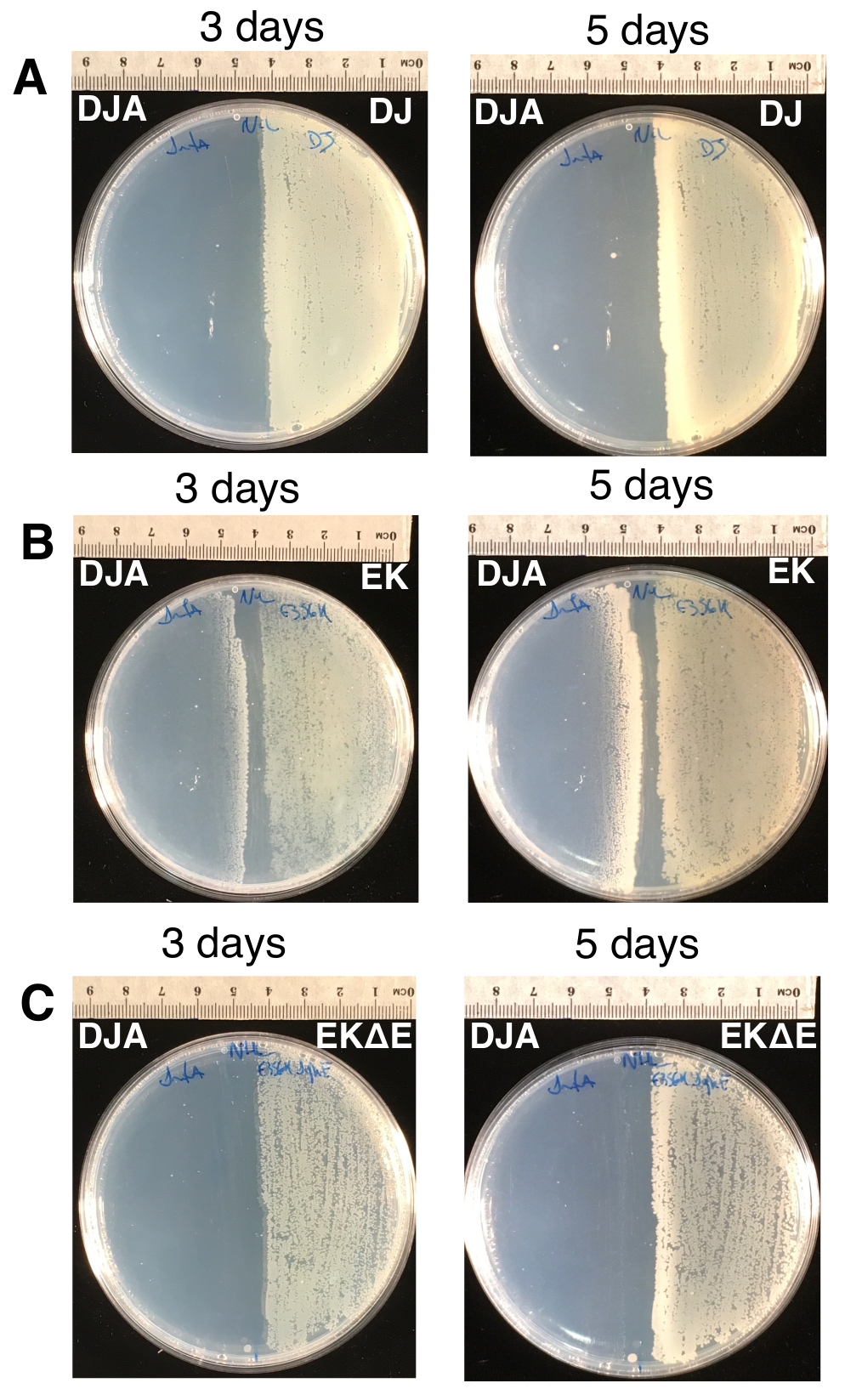

Supplement: S3 Fig — Cells were spread on opposite sides of an agar plate containing NIL media with 2% sucrose but without fixed nitrogen. The nifA deletion (DJA) is unable to grow unless a source of fixed nitrogen is provided. When spread opposite to the wild type strain (DJ) the nifA deletion strain (DJA) was unable to grow (A). In contrast, DJA grew when spread opposite to the strain EK due to the diffusion of excreted ammonium (B). When the glnE gene is deleted in the nifAE356K background (strain EKΔE), ammonium excretion is impaired and so is growth of the nifA deletion strain (C). (TIF) [file pgen.1009617.s003.tif]

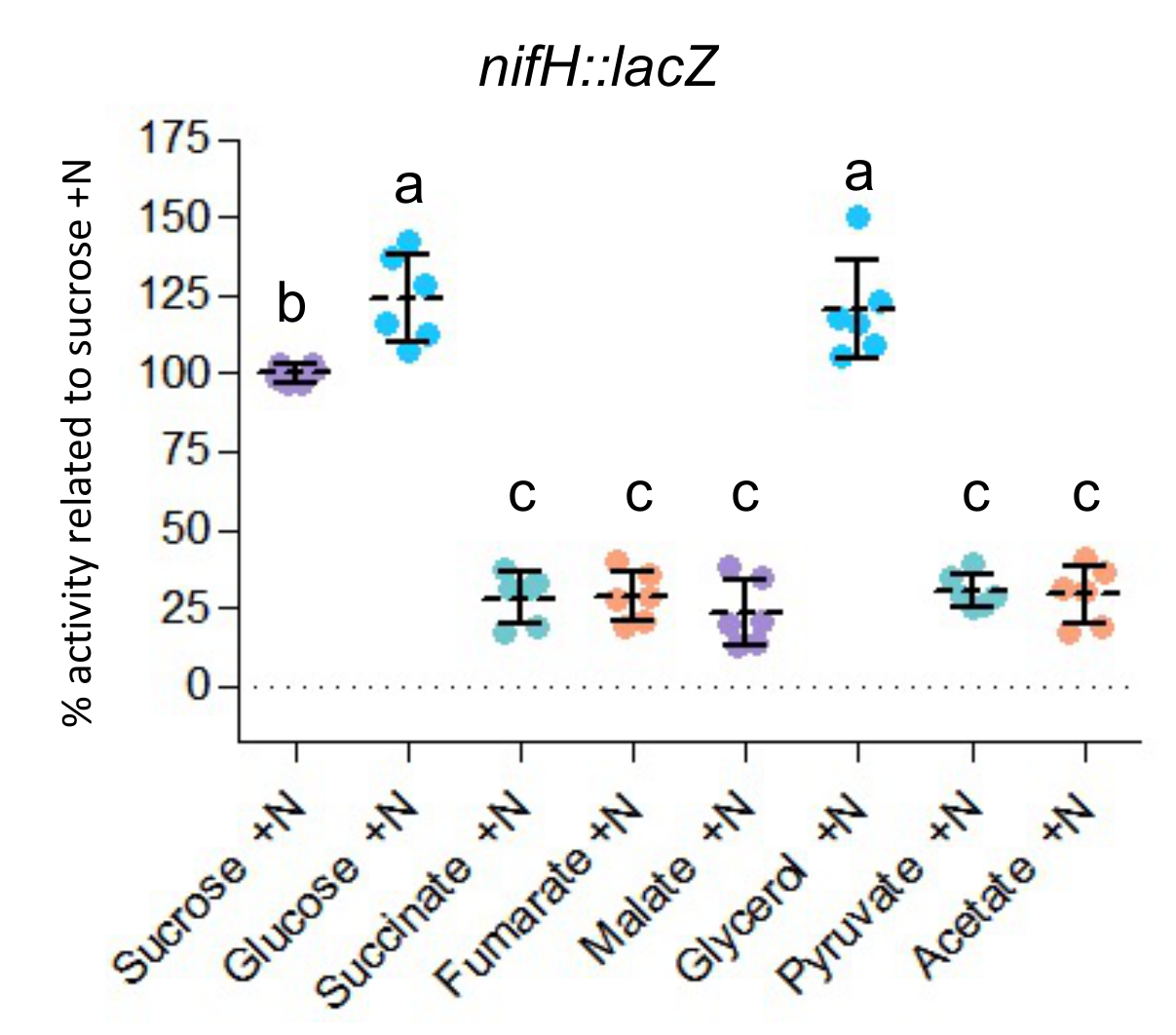

Supplement: S4 Fig — Cell suspensions (O.D600 = 0.1) were spotted as 10 μL drops (triplicate) on minimal solid media plates supplemented with 10 mM NH4Cl and the carbon sources indicated. After 18–36 hours incubation at 30°C, the grown bacterial biomass from triplicate spots were pooled and resuspended in 1 mL of PBS buffer. The β-galactosidase activity was performed using 100 μL of the PBS resuspended cells. Data is relative to the maximum level of detected activity in sucrose, calculated from specific β-galactosidase activity (1287.08 ±242.76 nmol ONP. mg protein-1. min-1). The data is representative from 3 independent experiments performed in technical duplicate. Plots followed by different letters are statistically different according to ANOVA with post-hoc Tukey’s HSD. (TIF) [file pgen.1009617.s004.tif]

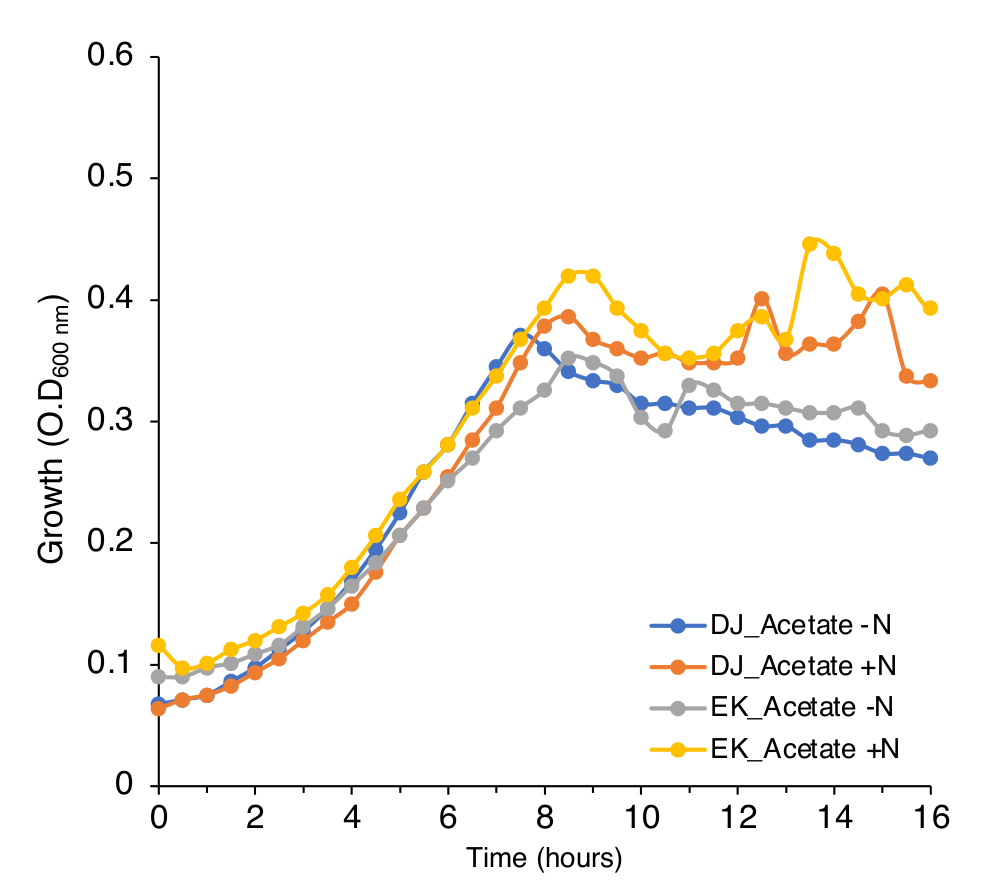

Supplement: S5 Fig — Cells were grown in MBB media supplemented with 30 mM acetate as carbon source without added ammonium (-N) or with 10 mM ammonium chloride (+N). Growth was assayed as described in S1 Fig. (TIF) [file pgen.1009617.s005.tif]

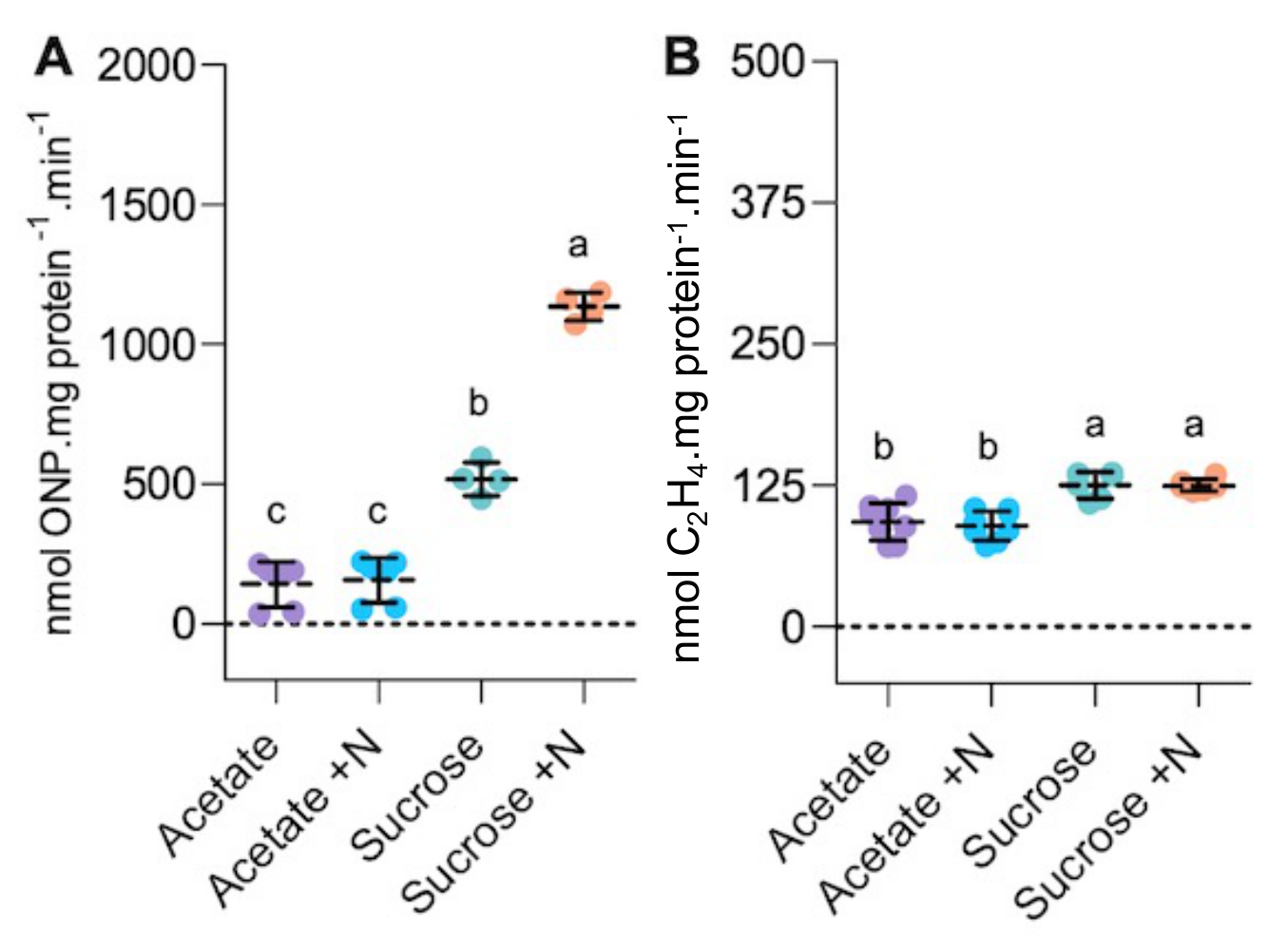

Supplement: S6 Fig — The nifL disrupted strain (AZBB163) was modified to encode a nifH::lacZ fusion (strain 163HZ) allowing ready comparison of nitrogenase expression in (A) and nitrogenase activity in (B). Plots followed by different letters are statistically different according to ANOVA with post-hoc Tukey’s HSD. (TIF) [file pgen.1009617.s006.tif]

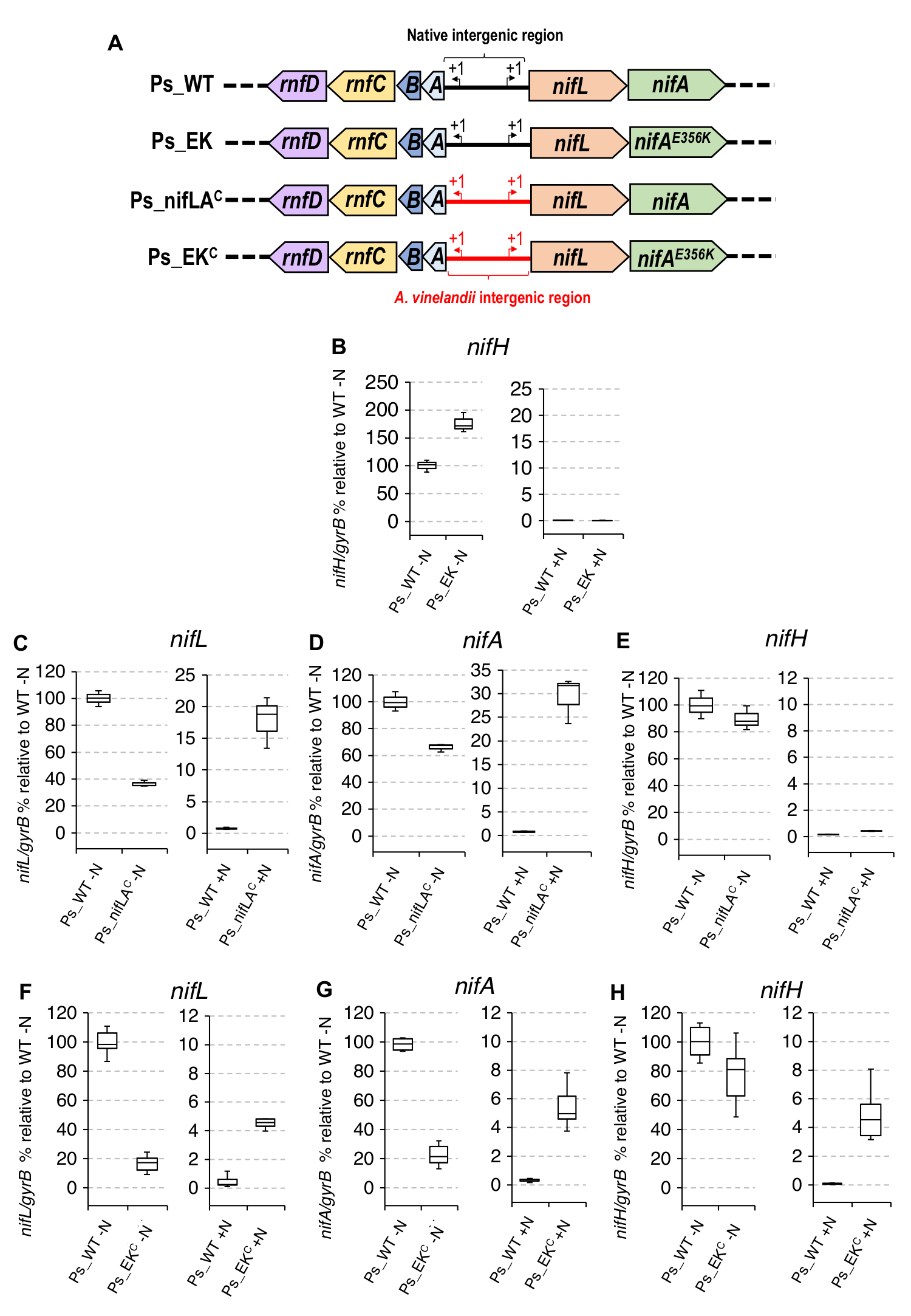

Supplement: S7 Fig — (A) Diagram depicting the genotypes of the P. stutzeri strains analysed. Drawings are not to scale. (B) nifH transcripts in the strain Ps_EK compared to the wild type (Ps_WT). (C-E) levels of nifL, nifA and nifH transcripts in the strain Ps_nifLAC compared to Ps_WT. (F-H) levels of nifL, nifA and nifH transcripts in the strain Ps_EKC compared to Ps_WT. Strains were grown under diazotrophic conditions (-N) or in the presence of excess fixed nitrogen (5 mM NH4Cl, +N). In each case the data is relative to the maximum level of detected transcripts under fully derepressing conditions (Ps_WT -N) estimated from absolute quantification. (TIF) [file pgen.1009617.s007.tif]

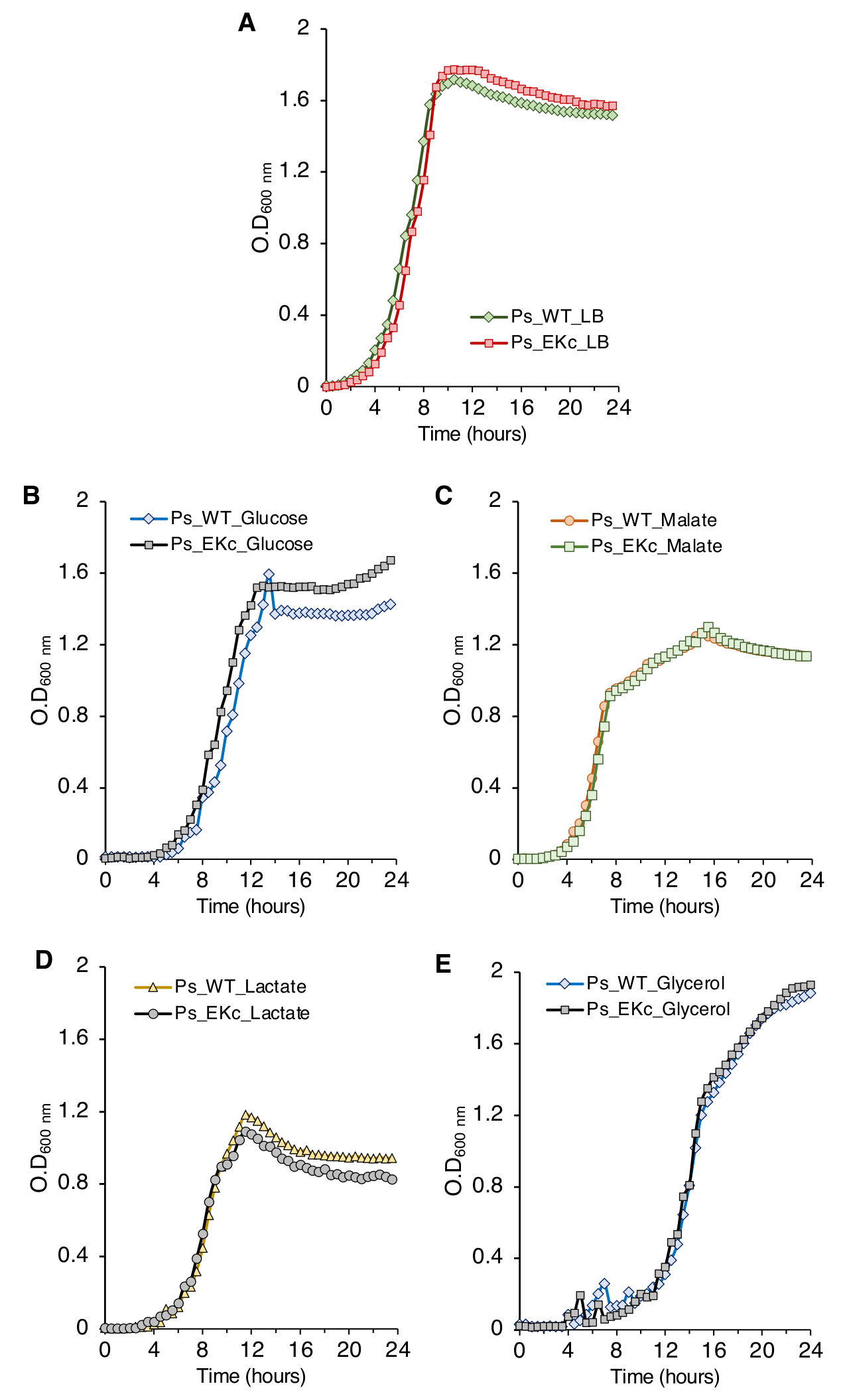

Supplement: S8 Fig — Growth as assayed in LB media (A) or in UMS-PS medium supplemented with 30 mM glucose (B), 45 mM malate (C), 60 mM lactate (D) or in 60 mM glycerol (D). In (B-D) the nitrogen source used was 5 mM NH4Cl. (TIF) [file pgen.1009617.s008.tif]

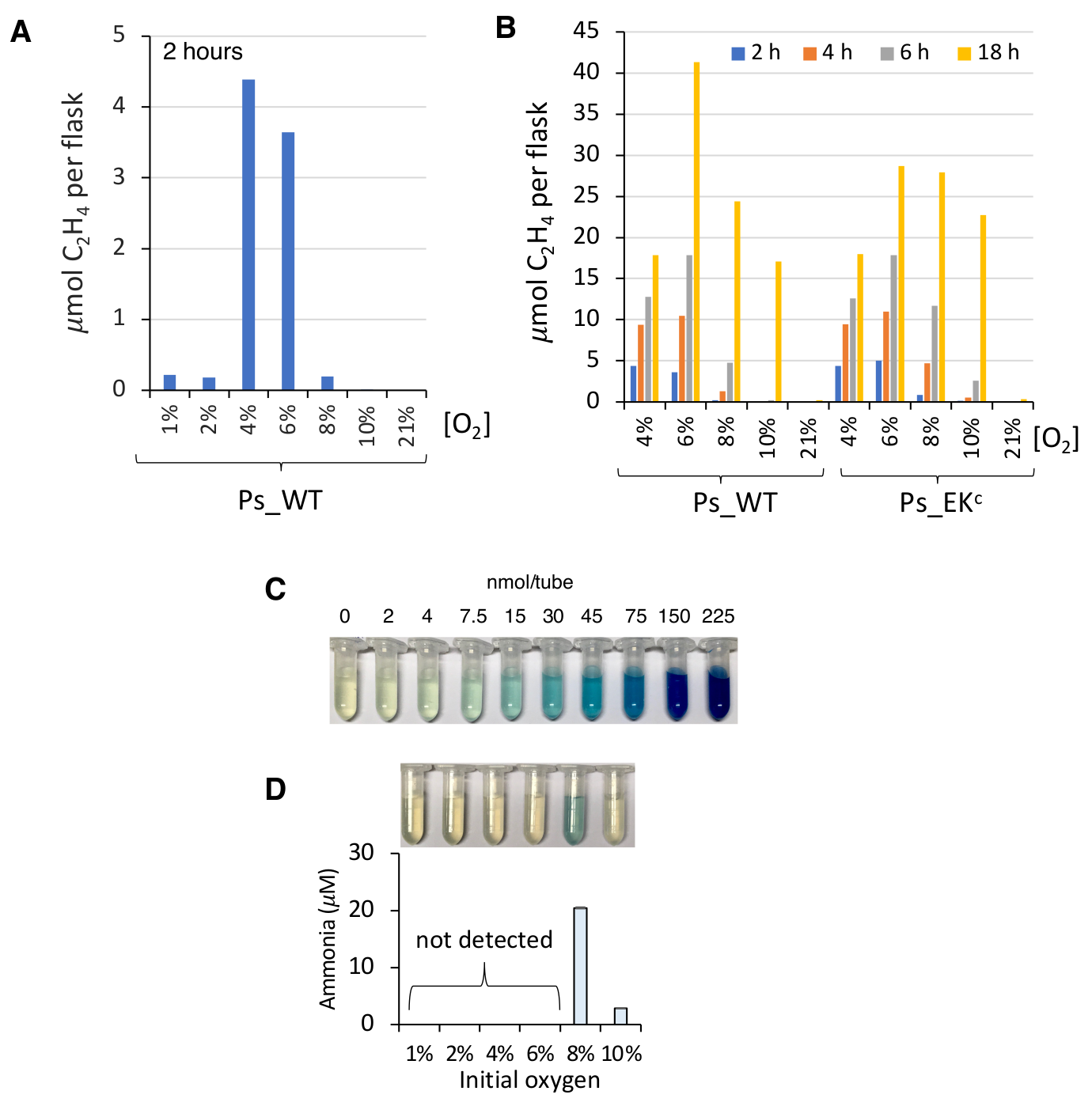

Supplement: S9 Fig — (A) Acetylene reduction assay of P. stutzeri A1501 (wild type, Ps_WT) grown under different initial oxygen concentrations in the gas phase of batch cultures. Typical specific activities under 4% O2 ranged from 18–24 nmol C2H4.mg protein-1.min-1. (B) Evaluation of the oxygen tolerance for detection of acetylene reduction upon longer incubation times for the P.stutzeri A1501 (Ps_WT) and nifA-E356K mutant (Ps_EKC). Ethylene (C2H4) was quantified 2 hours (blue bars), 4 hours (orange bars), 6 hours (green bars) and 18 hours (yellow bars) after acetylene injection. (C) Calibration curve for ammonia quantification by the indophenol method used to quantify ammonia in the supernatant of cultures in (D), where the ammonia excretion profile at defined initial oxygen concentrations are shown. Ammonia excretion was determined after 48 hours incubation. (TIF) [file pgen.1009617.s009.tif]
