# Supplementary material for: Disrupting hierarchical control of nitrogen fixation enables carbon-dependent regulation of ammonia excretion in soil diazotrophs
Source: PLoS Genet. 2021 Jun 10;17(6):e1009617. doi: 10.1371/journal.pgen.1009617 (PMC8219145; doi:10.1371/journal.pgen.1009617)
Supplement: S1 Table — (PDF) [file pgen.1009617.s010.pdf]

S1 Table. Strains used in this study

Strain	Relevant characteristic	Source
A. vinelandii		
DJ	ATCC BAA-1303. High frequency transforming variant of *A. vinelandii* UW generated in 1984 by Dennis Dean	[1]
DJA	DJ derived. 1401 bp deletion of *nifA* gene. Mo-Nif-, Rif^R	R.D. lab
DJH	DJ derived. *nifH::tetA*. Mo-Nif^-, Tc^R	This study
DJHZ	DJ derived. *nifH::lacZ* inserted into the *algU* locus. Tp^R	This study
EK	DJA derived. *nifA* deletion rescued by insertion of *nifA-E356K*	This study
EKH	EK derived. *ΔnifH::tetA* insertion into the native locus. Tc^R	This study
EKHZ	EK derived. *nifH::lacZ* fusion inserted into the *algU* locus. Tp^R	This study
EKΔE	EK derived. *ΔglnE::tmp* insertion into the native locus. Tp^R	This study
AZBB163	*nifL::Km*^{R}. Spontaneous mutant from AZBB150 resulting in the Nif^+ phenotype	[2]
163HZ	AZBB163 derived. *nifH::lacZ* inserted into the *algU* locus	This study
P. stutzeri		
A1501	CGMCC 0351, wild-type, isolated from rice roots inoculated with strain A15	[3,4]
Ps_*nifLA*^{C}	A1501 derived. *rnf-nifLA* intergenic region swapped by the reciprocal *A. vinelandii* region.	This study
Ps_EK^C-tetA	Ps_*nifLA*^{C} derived, but *Ps_nifAE356K* + *tetA* (Tc^R)	This study
Ps_EK^C	Ps_EK^C-tetA derived, but *tetA* cured with pMB2006	This study
Ps_EK	A1501 derived, Ps_*nifAE356K*	This study
E. coli		
ET8000	*rbs lacZ::*IS1 *gyrA hutC.* Used for β-galactosidase assays	[5]
NEB 5-alpha	Derivative of DH5α. T1 phage resistant and *endA* deficient. Used for cloning and plasmid maintenance	NEB
ST18	*E. coli* S17λpir Δ*hemA.* Used for conjugations	[6]

Abbreviations: Mo-Nif^-: no molybdenum-dependent nitrogenase activity; Rif: rifampicin, Km: kanamycin, Tc: tetracycline, Tp: trimethiorim, ^{R}: resistance

S1 Table References

1. Setubal JC, dos Santos P, Goldman BS, Ertesvåg H, Espin G, Rubio LM, et al. Genome Sequence of Azotobacter vinelandii, an Obligate Aerobe Specialized To Support Diverse Anaerobic Metabolic Processes. J Bacteriol. 2009;191: 4534–4545. doi:10.1128/JB.00504-09

2. Barney BM, Eberhart LJ, Ohlert JM, Knutson CM, Plunkett MH. Gene Deletions Resulting in Increased Nitrogen Release by Azotobacter vinelandii: Application of a Novel Nitrogen Biosensor. Appl Environ Microbiol. 2015;81: 4316–4328. doi:10.1128/AEM.00554-15

3. You C, Zhou F. Non-nodular endorhizospheric nitrogen fixation in wetland rice. Can J Microbiol. 1989;35: 403–408. doi:10.1139/m89-062

4. Vermeiren H, Willems A, Schoofs G, de Mot R, Keijers V, Hai W, et al. The rice inoculant strain Alcaligenes faecalis A15 is a nitrogen-fixing Pseudomonas stutzeri. Syst Appl Microbiol. 1999;22: 215–24. doi:10.1016/S0723-2020(99)80068-X

5. MacNeil T, Roberts GP, MacNeil D, Tyler B. The products of glnL and glnG are bifunctional regulatory proteins. Mol Gen Genet MGG. 1982;188: 325–333. doi:10.1007/BF00332696

6. Thoma S, Schobert M. An improved escherichia coli donor strain for diparental mating. FEMS Microbiol Lett. 2009;294: 127–132. doi:10.1111/j.1574-6968.2009.01556.x
