# Supplementary material for: Disrupting hierarchical control of nitrogen fixation enables carbon-dependent regulation of ammonia excretion in soil diazotrophs
Source: PLoS Genet. 2021 Jun 10;17(6):e1009617. doi: 10.1371/journal.pgen.1009617 (PMC8219145; doi:10.1371/journal.pgen.1009617)
Supplement: S2 Table — (PDF) [file pgen.1009617.s011.pdf]

S2 Table. Plasmids used in this study

Plasmid	Relevant characteristic	Source
pAAS1544	Cb^R. Derived from pPR54 [1] encoding *A. vinelandii* NifL (147-519) and NifA-E356K.	Adriano Stefanello
pPR34	Cb^R. *A. vinelandii nifLA* translated from the natural ribosome binding site of *nifL* in pT7-7	[1]
pPMA	Cb^R. Derived from pPR34, but encoding *nifA-E356K*	[2]
pRT22	Cm^R. *pnifH::lacZ* in pACYC184	[3]
pBlueScript SK II +	Cb^R. Cloning vector	[4]
pUC19	Cb^R. Cloning vector	[5]
pK18mobsacB Km	Km^R, Mob. Suicide vector for gene replacement. sacB gene for counter selection	[6]
pALMAR3	Tc^R. Source of tetracycline resistance gene (*tetA*)	[7]
pUC18T-mini-Tn7T-Tp	Tp^R. Source of the trimethoprim resistance gene (*tmp*)	[8]
pMB1724	Cb^R. 1893 bp PCR fragment corresponding to A. vinelandii *nifHD* region cloned into BamHI/HindIII sites of pBlueScript II +	This study
pMB1725	Cb^R. Tc^R. *tetA* gene amplified by PCR from pALMAR3 inserted into BglII and EcoRI sites of pMB1724	This study
pMB1804	Cb^R. Derived from pPR34 encoding *P. stutzeri* A1501 *nifL-nifA*	This study
pMB1805	Cb^R. Derived from pPR34 encoding *P. stutzeri* A1501 *nifL-nifA-E356K*	This study
pMB1806	Cb^R. Derived from pPR34 encoding *A. olearius* DQS-4 *nifL-nifA*	This study
pMB1807	Cb^R. Derived from pPR34 encoding *A. olearius* DQS-4 *nifL-nifA-E351K*	This study
pMB1816	Cb^R, Tp^R. *A. vinelandii nifH::lacZ* fusion flanked by homology regions for integration into *algU* genome locus	This study
pMB1840	Km^R. Tp^R. *A. vinelandii glnE* deletion. Fragments upstream (906 bp) and downstream (752 bp) of *glnE* were fused to the *tmp* gene, and inserted into pk18mobsacBKm cut with SmaI	This study
pMB2005	Km^R. The *rnf-nifLA* intergenic region (444 bp) from *A. vinelandii* was fused downstream to a fragment of the *P. stutzeri rnfAB* genes (1160 bp) and upstream to a fragment of the *P. stutzeri nifL* gene (1638 bp) and inserted into pk18mobsacBKm cut with SmaI	This study
pMB2006	Km^R. Ps-*nifLAE356K* fragment (2364 bp) from pMB1805 was fused to an 847 bp fragment downstream Ps-*nifA* and inserted into pk18mobsacBKm cut with SmaI. Construct to recover *tetA* from Ps_EK^C-*tetA* to generate Ps_EK^C	This study
pMB2007	Km^R, Tc^R. The plasmid pMB2006 was linearized by PCR and fused to a fragment encoding the *tetA* gene (1349 bp). Construct to generate Ps_EK^C-*tetA*	This study

Abbreviations: Cb: carbenicillin, Cm: chloramphenicol, Km: kanamycin, Tc: tetracycline, Tp: trimethiorim, ^{R}: resistance.

S2 Table References

1. Söderbäck E, Reyes-Ramirez F, Eydmann T, Austin S, Hill S, Dixon R. The redox- and fixed nitrogen-responsive regulatory protein NIFL from Azotobacter vinelandii comprises discrete flavin and nucleotide-binding domains. Mol Microbiol. 1998;28: 179–192. doi:10.1046/j.1365-2958.1998.00788.x
2. Reyes-Ramirez F, Little R, Dixon R. Mutant Forms of the Azotobacter vinelandii Transcriptional Activator NifA Resistant to Inhibition by the NifL Regulatory Protein. J Bacteriol. 2002;184: 6777–6785. doi:10.1128/JB.184.24.6777-6785.2002
3. Tuli R, Merrick MJ. Over-production and characterization of the nifA gene product of Klebsiella pneumoniae--the transcriptional activator of nif gene expression. J Gen Microbiol. 1988;134: 425–432. doi:10.1099/00221287-134-2-425
4. Alting-Mees MA, Short JM. pBluescript II: gene mapping vectors. Nucleic Acids Res. 1989;17: 9494. Available: http://www.ncbi.nlm.nih.gov/pubmed/2555794
5. Norrander J, Kempe T, Messing J. Construction of improved M13 vectors using oligodeoxynucleotide-directed mutagenesis. Gene. 1983;26: 101–106. doi:10.1016/0378-1119(83)90040-9
6. Schäfer A, Tauch A, Jäger W, Kalinowski J, Thierbach G, Pühler A. Small mobilizable multi-purpose cloning vectors derived from the Escherichia coli plasmids pK18 and pK19: Selection of defined deletions in the chromosome of Corynebacterium glutamicum. Gene. 1994;145: 69–73. doi:10.1016/0378-1119(94)90324-7
7. Malone JG, Jaeger T, Spangler C, Ritz D, Spang A, Arrieumerlou C, et al. YfiBNR mediates cyclic di-GMP dependent small colony variant formation and persistence in Pseudomonas aeruginosa. PLoS Pathog. 2010;6. doi:10.1371/journal.ppat.1000804
8. Choi K-H, Schweizer HP. mini-Tn7 insertion in bacteria with single attTn7 sites: example Pseudomonas aeruginosa. Nat Protoc. 2006;1: 153–161. doi:10.1038/nprot.2006.24
