# Supplementary material for: Disrupting hierarchical control of nitrogen fixation enables carbon-dependent regulation of ammonia excretion in soil diazotrophs
Source: PLoS Genet. 2021 Jun 10;17(6):e1009617. doi: 10.1371/journal.pgen.1009617 (PMC8219145; doi:10.1371/journal.pgen.1009617)
Supplement: S3 Table — (PDF) [file pgen.1009617.s012.pdf]

S3 Table. Primers used in this study

ID	Sequence 5’ -> 3’	Application
AAS-3	**GAATGCCCATGAATGCAACCAT**	Amplification of Av-*nifA* for genotyping
AAS-55	**TCAGATCTTGCGCATGTGGATGT**	
2-43	**AAAAGGATCCCTCATGTTTGACAGCTTATC**	Amplifies the *tetA* cassette from pALMAR3 for Av-*nifH* insertion
2-44	**AAAAGAATTCTTGATTGGCTCCAATTCTTG**	
2-45	**AAAAGGATCCGTCACTGAACTCTCCTGCTGAGG**	Amplifies 1874 pb of Av-*nifHD* (position 136301 – 138174)
2-46	**AAATAAGCTTTCCACTTCGTCGATCAGTTTGGCG**	
5-49	**GAGTTGTTCGCTTTCTACGGAATCATTGGTGATTTCGG**	Amplifies 409 bp corresponding to the Av-*nifH* promoter (position 136401 – 136809)
5-50	**TGTAAAACGACGGGATCCCCGGACTTACCGATACCACC**	
5-46	**GGGGATCCCGTCGTTTTACAACGTCGTG**	Amplifies the *lacZ* gene (3057 bp) and from pRT22
5-464	**CTAGCGGTTATTATTATTTTTGACACCAGACCAACTG**	
7-28	**CGAATTCGAGCTCGGTACCCCCATGGAAACGGTGGGGAACTG**	Amplifies 906 bp upstream Av-*glnE* (position 4547284 – 4548189)
7-29	**AATTATCCATACTGTCGCCGAGCGTGCC**	
7-30	**CGGCGACAGTATGGATAATTCACGAACC**	Amplifies a 631 bp fragment from pUC18T-mini-Tn7T-Tp encoding to the *tmp* resistance gene
7-31	**CCGGCTGCTTTAATAACCGCTAGATAATTCTTAG**	
7-32	**GCGGTTATTAAAGCAGCCGGGCGTGGTG**	Amplifies 752 bp downstream Av-*glnE* (position 4543778 – 45444529)
7-33	**GTCGACTCTAGAGGATCCCCGCCTTCCGGATCGAGCAGC**	
3-75	**AAGCTTATCGATGATAAGCTG**	Amplification of 2421 bp from pR34 excluding Av-*nifLA*
4-14	**ATGGTGCCTCGTCTATCCGAA**	
4-17	**GGAAGCTCGCATGAACGCCACATTCGCC**	Amplification of Ps-nifA for constructing pMB1804
4-18	**CGATAAGCTTTCAGATCTTGCGCATATGAATG**	
4-19	**GAGGCACCATATGGCTTTGCAACGGATACC**	Amplification of Ps-nifL for constructing pMB1804
4-20	**TGGCGTTCATGCGAGCTTCCCCTGTCAG**	
4-21	**CGCGACCTGAAGCATGAGGTGGAG**	Introducing E356K mutation into Ps-nifA
4-22	**CGCGACCTGAAGCATGAGGTGGAG**	
3-74	**CAGCTTATCATCGATAAGCTTTCAGATCTGCCGCACCTTGAT**	Amplification of Ao-nifA for constructing pMB1806
3-74B	**GACCTGGAGGGCGGTCGATGAGCGCGGCCGGTCCGATG**	
4-12	**CATCGGACCGGCCGCGCTCATCGACCGCCCTCCAGGTC**	Amplification of Ao-nifL for constructing pMB1806
4-13	**TTCGGATAGACGAGGCACCATATGGGCGCTGTCGCCGACG**	
2-37	**ACCAACCGCGACCTCAAGCTCGAGGTCGAAGCG**	Introducing E351K mutation into Ao-nifA
2-38	**CGCTTCGACCTCGAGCTTGAGGTCGCGGTTGGT**	
8-1	**TGATTACGAATTCGAGCTCGGTACCCTCAGTCTTCGGTTTCCGCCGCT**	Amplification of 847 bp downstream Ps-*nifA*
8-2	**GCGCAAGATCTGAACGACCCGCCCG**	
8-3	**TCGGGCGGGTCGTTCAGATCTTGCG**	Amplification of Ps-*nifLAE356K* (2364 bp) fragment from pMB1805
8-11b	**CTCTAGAGGATCCCCACCGGGCTGCGCCAGCAA**	

8-13	ACATGAGCCCGGGTCGGGC	Linearizes the pMB2006 by PCR for *tetA* insertion
8-14	CCAATCAAAGTCGGGCGGGTCG	
8-15	GCCCGACTTTGATTGGCTCCAATTCTTGGAGTG	Amplifies the *tetA* cassette from pALMAR3 for insertion into pMB2006
8-16	GACCCGGGCTCATGTTTGACAGCTTATCATCGATTAGC	
8-17	TCGAGCTCGGTACCCTCAGGCGGCCCT	Amplification of Ps-*rnfAB* (1160bp)
8-18	GAGGTCGCTTATGGAATATGCGCTGTTTCTGATCG	
8-19	CATATTCCATAAGCGACCTCACCTGCT	Amplification of the *A. vinelandii rnf-nifLA* intergenic region (444 bp)
8-20	AAAGCCATGCTGTGCCTCGTCTATCCA	
8-21	GCACAGCATGGCTTTGCAACGGATACCG	Amplification of Ps-*nifL* (1638 bp)
8-22	GACTCTAGAGGATCCCCTCAGCTGGCCGAGAAGGG	
M13F (-47)	CGCCAGGGTTTTCCCAGTCACGAC	Sequencing and PCR linearization of constructs
M13R (-48)	AGCGGATAACAATTTCACACAGGA	
Av-RTgyrBF	CAAGAAGCACAAGGTGACGA	RT-qPCR *A. vinelandii gyrB*
Av-RTgyrBR	TGGTCTGCGAACTGAACTTG	
AvRTnifH-3F	CAGCCCTGGCTGAGATGGG	RT-qPCR *A. vinelandii nifH*
AvRTnifH-3R	ATGGTGTTCTGGGCCTTGGA	
Av-RTnifLF	CTGCTGACCATCAACGACAT	RT-qPCR *A. vinelandii nifL*
Av-RTnifLR	ATGCCTTCCAGCAGCTCTT	
Av-RTnifAF	GCAAGTACGGCTTCGAGAAC	RT-qPCR *A. vinelandii nifA*
Av-RTnifAR	GTACGGTGCTGTTCCACTTG	
Ps-RTgyrB2F	AAACCATCCGCCGAGACCTT	RT-qPCR *P. stutzeri gyrB*
Ps-RTgyrB2R	CCCACCCCGGAGTTGAGAAA	
Ps-RTnifH1F	CCACGACCCAGAACCTCGTG	RT-qPCR *P. stutzeri nifH*
Ps-RTnifH1R	GAGTGCAGGATCAGGCGAGT	
Ps-RTnifL2F	AGGACATGCACATGACCCAGG	RT-qPCR *P. stutzeri nifL*
Ps-RTnifL2R	CACGGCTTGCTGGAACACTT	
Ps-RTnifA7F	TCCTCAAGCATGGCAACAGC	RT-qPCR *P. stutzeri nifA*
Ps-RTnifA7R	GAAGGGCAGGTCCATGTCGTA	
