# Supplementary material for: Disrupting hierarchical control of nitrogen fixation enables carbon-dependent regulation of ammonia excretion in soil diazotrophs
Source: PLoS Genet. 2021 Jun 10;17(6):e1009617. doi: 10.1371/journal.pgen.1009617 (PMC8219145; doi:10.1371/journal.pgen.1009617)
Supplement: Attachment — Submitted filename: Response to reviewers_PlosGen_revised_final.docx [file pgen.1009617.s013.docx]

*We thank the reviewers for their constructive comments on our manuscript. Our point-by-point answers are presented in* blue *text below.*

**Comments to the Authors:**

Reviewer #1: This is a typical Dixon paper, thoroughly thought through work with a clear message. Overall, the work behind is technically not very complicated, but the outcome is striking: the single amino acid substitution on NifA gives rise to an ammonium excretion phenotype of the respective Azotobacter strain, and this property can be transferred to other N2-fixing Proteobacteria (with potential agricultural impact in synthetic plant -bacterial communities)

The authors provide all the necessary data to explain this phenotype, including the repression of GS activity through adenylylation. Therefore, the conclusions are backed up by the data.

We thank the referee for his/her positive evaluation of our manuscript. We are delighted to hear that our manuscript delivers a *“thoroughly thought through”* message.

There are only a few minor suggestions to improve the accessibility of the paper: it would be good to have the strain abbreviations included in the main text and not only in supplemental table:

We agree that this may improve the manuscript and have now included the strain abbreviations where relevant.

Furthermore, the model resented in For 6 could be further improved by indicating the activity state of NifA (to easier distinguish the active state in B and F from the inactive states.

A similar request was also made by reviewer 2. In response to these, we have now amended Fig. 6 to indicate the activity state of NifA in panels B and F.

Reviewer #2: Nitrogen fixation is an energy-expensive process. For the biological reduction of nitrogen gas into ammonia, the enzyme nitrogenase utilizes 8 equivalents of ATP in each reaction cycle. The process is quite complex involved several gene products dedicated to the maturation of cofactors and enzyme components. Thus, tight regulation of this process guarantees that nitrogen fixation is only employed under conditions when no other fixed nitrogen source is available and when cells have enough nutrients to sustain this energy-demanding process. The two-component NifA-NifL transcriptional regulatory system guarantees that nitrogen fixation is activated when the right physiological and nutritional conditions are met. Notably, the NifA-NifL control guarantees that no excess of ammonia is produced, maximizing the efficiency of this system regarding the energetic toll caused by nitrogen fixation.
In previous work, the corresponding author Dixon and collaborators reported the identification of the NifA E356K variant, which was insensitive to nitrogen regulation. The E356 residue is contained within the AAA+ domain, and the screening of suppressor mutations revealed the importance of GAF and AAA+ interdomain communication for this regulatory sensing. In this study, Batista et al. further characterized this variant (NifA E356K) that bypasses the nitrogen sensing while maintaining signaling responses via 2OG.

This study builds on previous scientific contributions by Dixon and others. It provides insight into potential strategies on how to engineer microbes with expanded traits suitable for biotechnological applications in agriculture. Although the identification of this variant has been known for over 15 years, this report provides a mechanistic model for this interesting phenotype that uncouples to two regulatory features of NifA, namely nitrogen and carbon sensing.

Uncoupling nitrogen fixation from ammonia assimilation is a topic that merits investigation since it is one of the factors that limit the wide use of diazotrophs as natural fertilizers. The experimental design to characterize this variant was sounded and provided a mechanistic model for the dual regulation based on nitrogen and carbon source availability.

We are pleased to hear the positive feedback from Reviewer 2.

I do not have any major comments in this submission, but I would like to provide a general suggestion for revising figure 6 and 3 so the information presented in the figure could be a little more self-explanatory. As presented, the reader needs to dig into the figure legend. Certainly, this comment does not preclude the publication of this study.

Figure 3 panels shaded in green and yellow could be annotated with +N and -N.

We believe the reviewer was referring to Fig. 2 rather than Fig.3.We have modified Fig. 2 accordingly. Please note that to address questioning from reviewer 3, panel F from Fig. 2 was also modified. See the details below in the response to reviewer 3.

For figure 6 the suggestion is to indicate conditions for N limiting and N excess and indicate that NifA, when dissociated from the complex, is in its active form promoting transcription nitrogen fixation.

A similar suggestion was made by reviewer 1. The figure has now been modified to incorporate the changes suggested by both reviewers.

Lastly, it would be good to address the literature or experimental evidence demonstrating the binding of GlnK to NifL shown in panel F. While it has been reported that GlnK directly binds to NifL, prior literature indicated that such event occurs during the formation of a ternary complex with NifA.

We are not aware of any prior literature indicating that the GlnK-NifL interaction only occurs in the ternary complex with NifA. Two independent studies demonstrated interaction between NifL and GlnK *in vitro* and *in vivo* in the absence of NifA. The study in ref. 12 demonstrated that GlnK can be pulled-down using Av-NifL-6xHis immobilized on Ni-NTA-magnetic agarose beads. In addition, *in vivo* evidence for a binary interaction between Av-GlnK and Av-NifL has also been obtained by yeast two-hybrid assays (ref. 13).

Reviewer #3: Overall, this is a well written and very interesting piece of work on the regulation of interaction between NifL and its cognate sigma factor NifA. It shows convincingly that a single point mutation E356K enables NifA to escape from regulation of a ternary complex with NifL and GlnK so long as 2-oxoglutarate is at sufficient concentration to bind to the GAF domain of NifA.

Reviewer specific comments

You have used the term “synthetic symbiosis” multiple times throughout the paper but fail to define what you mean by this. I assume you mean engineering control over nitrogen fixation and ammonia secretion in cereal associative bacteria? Maybe you could introduce the term in the Line 65 paragraph in the introduction.

This is now added at the end of the introduction, where the term “synthetic symbiosis” appears for the first time.

Plant root exudates are known to be rich in dicarboxylates and other carboxylic acids, they generally do not secrete sucrose for example. You have shown in this paper that when your bacteria are grown on various dicarboxylates as sole carbon sources, NifA-E356K remains sensitive to regulation by the nitrogen status (Fig S4). This directly contradicts your claim that NifA-E356K could be useful for engineering N-insensitive diazotrophs for agricultural use. The authors should provide some acknowledgement/interpretation of this.

In Fig. S4, we demonstrate that the ability of NifA-E356K to escape NifL repression in excess nitrogen is not exclusively dependant on sucrose. In simpler carbohydrates, such as glucose or in a polyol such as glycerol, NifAE356K activity is even higher than in sucrose. Given the dynamic nature of root exudates in different crops, their precise composition is difficult to define. Therefore, we do not think that the supposed absence of sucrose in root exudates contradicts our hypothesis, but we have nevertheless acknowledged the referee’s point in the discussion by inserting a new sentence (lines 388-390):

*“However, given that the composition of root exudates is dynamic and difficult to define, engineering of the partner plant to provide a favourable carbon source may also be required”*

Specific comments

Line 61. The binding of 2-ketoglutarate by the PAS domain of NifA is such an important point that I want to see a primary reference where the measurements are reported and not a review

We believe this is a misunderstanding. The primary reference where binding of 2-oxoglutarate to the NifA GAF domain was demonstrated had already been cited in the submitted version (originally as ref. 14, now ref 15). This paper is certainly not a review. However, we now have included a reference to an additional study where the role of 2-oxoglutarate was first suggested (ref. 14 in the revised version).

Page9 bottom and Figure S4. So growth on mono or di-carboxylic acids does not support such high activation of the integrated nifH-lacZ fusion. The implication is that sugars produce a higher level of 2-ketoglutarate (and this is measured), which is contrary to the simplistic expectation that solutes dependent on flux through the TCA-cycle will produce more 2-ketoglutarate. However, this may of course be a consequence of steady state levels of intermediates in the TCA-cycle being lower when growing on TCA-cycle intermediates due to a high rate of flux. I would be interested to know what genome scale models of A. vinelandii predict for intermediates of the TCA-cycle when grown on sugars or organic acids but this is clearly outside the scope of this project. However, as 2-ketoglutarate levels been measured on the different growth substrates (Fig 3) and they are higher for sugars this is consistent high TCA-cycle flux reducing the steady state concentration of 2-ketoglutarate. Perhaps a comment about flux through the TCA cycle is likely to be higher during growth on organic acids and this is likely to reduce 2-oxoglutarate levels would be helpful.

We thank the reviewer for this insightful comment. We agree that metabolic flux analysis would be very interesting to better understand how different carbon sources influence the steady state levels of 2-oxoglutarate. There are already metabolic flux models available for *A. vinelandii* (see Castillo et al 2013, ***Process Biochemistry***, Volume 48, Issue 7, Pages 995-1003 <https://doi.org/10.1016/j.procbio.2013.04.014> and Wu et al 2019, **Sci Rep**, 9:13209, <http://dx.doi.org/10.1038/s41598-019-49717-6>). In these studies, the analysis was only performed in glucose aiming to evaluate the effects of oxygen and nitrogen variation in the metabolic flux, respectively. As the growth conditions in both mentioned studies were different from the conditions used in our study, we believe that extrapolation of their models is very difficult to make and therefore, we would prefer not to make any further assumptions in this regard. Finally, as pointed out by the reviewer this is outside the scope of this manuscript.

Lines 253 There is an alignment of NifA sequences in Figure 4 and this includes alpha, beta, gamma and zeta-proteobacteria but data is only given for Beta and Gamma proteobacteria, which is understandable. However, do the examples of alpha and zeta-proteobacteria have nifL?

We agree with the reviewer that this was not completely clear in the submitted version. The given examples of alpha and zeta-proteobacteria used in the referred alignment do encode NifL. We have modified the legend of Fig. 4 to clarify this:

“*(B) Alignment of residues close to E356 (black arrow) in the central AAA+ domain of NifA proteins regulated by NifL.*”

Is NifL the most common system for O2 sensing in bacteria other than gamma-proteobacteria or is it intrinsic O2 sensitivity of NifA? In other words, I would a simple but clear statement of the relative abundance of NifL in the groups of proteobacteria. This is not a trick question it just helps the reader to see how widespread the system is even if it is quite rare in some groups of bacteria.

We have discussed the distribution of NifL-NifA across Proteobacteria in a recent review (ref. 3). We have now amended the text (Lines 254-256) to emphasize that although regulation by NifL is not the most prevalent mechanism for controlling NifA activity in bacteria, the *nifL-nifA* operon is widely distributed in Proteobacteria [and the glutamate residue at position 356 in *A. vinelandii* NifA is highly conserved.

Lines 288-295. I agree with the summary of the authors on the role of the E356K mutation. However, the rates of acetylene reduction and ammonia release, even on lactate, are extremely low for the engineered P. stuzeri. They are approximately 1% of the rates seen for A. vinelandii. From Figure S7 I see there is a very small overall escape from N-regulation in the engineered strain so essentially P. stuzeri still manages to maintain pretty good N-control of fixation in spite of the weak escape due to E356K and use of the A. vinelandii promoter. This is further discussed by the authors in the discussion.

Fig. S7 demonstrates (Panel H) that in strain Ps_EK^C^, the levels of *nifH* under excess nitrogen (+N) are approximately 5% of the maximum level detected under nitrogen limitation (-N, maximum activation). Please note that the *nifL* and *nifA* transcripts in +N (Panels F and G) are also in the same range (approximately 5% of the maximum detected in -N). Therefore, Ps_NifA-E356K is fully competent to escape nitrogen regulation. In addition, note that when expressed in *E. coli* (Fig. 4) the Ps-NifA-E356K is able to fully escape nitrogen regulation.

Lines 360-362. You have shown that you do get some ammonia secretion when NifA-E356K is expressed in the glnE mutant which is unable to switch off ammonia assimilation by GS (Fig 2D-F). Thus, it is not necessary to inhibit GS to drive ammonia secretion, but it certainly enhances it.

We have reviewed our calculation spreadsheets and realised that the culture media background interference had not been subtracted from the data plotted in Fig. 2F. We have revisited our calculations and now can confirm that no ammonia excretion is detected in the strain EKΔE, as also suggested by the qualitative cross-feeding assay in Fig. S3. Therefore, we believe that the statement in lines 367-370 does not require modification. As a consequence of our revised calculations, Fig. 2F is now modified as below:

Fig. 2F is now plotted on a linear scale since null values cannot be plotted on a log scale.

Line 400. It might be worth mentioning here that other studies routinely assay nitrogenase activity in P. stuzeri with a starting O2 concentration of 1% (see Setten et al., 2013 & Ryu et al 2020). From the methods, your assays are done at 4% or 10% starting O2 (I am unsure which has been used). It is worth acknowledging here that you may not be observing optimal rates of nitrogen fixation.

We did not mention in the original submitted version that we performed oxygen titrations to define the optimal initial oxygen concentration for nitrogenase activity and ammonia excretion under our experimental conditions. We have now amended the methods and the data is now presented in supplementary Fig. S9. Based on our titrations, rapid de-repression of nitrogenase for transcript detection was performed starting with 4% O2 in the gas phase (Fig. 5B) following 4 hours incubation. Regardless of the apparent optimal rate of acetylene reduction under 4% O2, ammonia excretion, was not detected under these conditions. Therefore, ammonia excretion experiments using *P. stutzeri* Ps_EK^C^ were performed under 8% O2 (Fig. 5D). Our results suggest that prior to ammonia excretion, the organism needs to trigger uncoupling of ammonia assimilation via feedback inhibition of GS activity. We hypothesise that if nitrogenase activity is limited by reduced ATP levels and electron flow under the limiting oxygen conditions that arise during a longer period of incubation, internal levels of ammonia will not build up at sufficient concentrations to prompt the feedback mechanism that triggers ammonia excretion.

The methods (lines 597-601) were modified as follows:

*“Given the microaerobic lifestyle of P. stutzeri, the flasks were prepared at defined initial oxygen concentrations of 4% or 8% in the gas phase of the flasks, based upon an O_2_ titration to define appropriate conditions for rapid nitrogenase de-repression or ammonia excretion, respectively (Fig. S9)”.*

Line 429. Is this data for comparison of phenotypes in NIL and MBB media provided? If so, could cite it here.

Before implementing the use of MBB media, we compared diazotrophic growth profiles in 2% sucrose as in the figure below. The data in the left panel is the same as presented in Figure S1.

The **diazotrophic** growth profiles were similar between both media and the ammonia excretion levels were identical. However, MBB medium prevents precipitation and is thus more suitable for growth experiments in a plate reader

Line 484. Isothermal assembly? I see you mention in Line 547 that this is done with HiFi mastermix. Best to give this information when the term first appears or before-hand.

We thank the reviewer for this observation. We have now moved the section “Recombinant DNA work” up to lines 470-477 so that the explanation of how the Isothermal assembly is done appears before the technique is mentioned in the subsequent plasmid construction sections.

Line 555. You use the term “in brief” many times in the methods. I think this is redundant. Consider deleting.

We apologise for this. This in now amended in the revised version.

Line 595. You state that P. stuzeri was assayed for nitrogenase activity at after adjusting the initial O2 concentration to 4% or 8%. It is not clear in your figures which was used.

We agree that this may not have been very clear. We have now indicated the oxygen regimen used in the relevant figure legend and amended the methods accordingly by adding more details in the new supplementary figure (Fig. S9).
